# A Manual of the Practice of Medicine

**Published:** 1891-09

**Authors:** 


A Manual of the Practice of Medicine. By Frederick Taylor, M. D., F. R. C. P., Physician to, and Lecturer on Medicine, at Guy's Hospital; Physician to the Evelina Hospital for Sick Children; Examiner in Materia Medica and Pharmaceutical Chemistry, at the University of London. With illustrations. Philadelphia: P. Blakiston, Son & Co., 1012 Walnut street. 1890.

This is a very good text-book, and deserves a place in the front rank, beside Roberts and Strümpel. The section on the Methods of Procedure in Examination of the Nervous System is especially good.

In the remarks on Physical Diagnosis there is one statement that is misleading, viz.: "In certain parts of the chest the vesicular murmur gives place to a sound having the character which will be presently described as those of bronchial breathing. These parts are the upper end of the sternum, the first costal cartilages at their junction with the sternum, and a diamond-shaped span at the back in the middle line, including the seventh cervical and first dorsal spines. Elsewhere the vesicular murmur is always present, as long as the lung is healthy and the air passages pervious." A grave error is here made in not stating that the breathing in the right infra-clavicular region is much more bronchial in character than that in the left, being of a longer expiratory sound, and of higher pitch. This is an important point, as, if not thus instructed, a young practitioner might make the serious mistake of diagnosticating an infiltrated right apex, merely from the normal physical signs, especially as the percussion note in that region is also of a higher pitch than in the corresponding area on the left side.

In the section devoted to the Diseases of the Stomach no mention is made of the use of the stomach-tube for purposes of diagnosis. This is a serious omission, for the tube is of even greater value as a means of diagnosis than as a means of treatment.

Another omission of serious import is that of the therapeutic uses of inhalations of oxygen gas, as in the various forms of anemia, in pneumonia, and in edema of the lungs.

The section devoted to Diseases of the Heart is very clear and concise.

With the few exceptions noted above, the book is, on the whole, to be highly recommended. De L. R.

